# Serum neuron-specific enolase as early predictor of outcome after in-hospital cardiac arrest: a cohort study

**DOI:** 10.1186/cc5046

**Published:** 2006-09-15

**Authors:** Tatiana H Rech, Silvia Regina Rios Vieira, Fabiano Nagel, Janete Salles Brauner, Rosana Scalco

**Affiliations:** 1 Serviço de Medicina Intensiva, Hospital de Clínicas de Porto Alegre, Rua Ramiro Barcelos, 2350. Largo Eduardo Z. Faraco, Porto Alegre, RS, 90035-903, Brazil; 2 Serviço de Medicina Intensiva, Complexo Hospitalar Santa Casa de Misericórdia de Porto Alegre, Rua Prof. Anes Dias, 295. Porto Alegre, RS, 90020-090, Brazil; 3 Serviço de Patologia Clínica, Hospital de Clínicas de Porto Alegre, Rua Ramiro Barcelos, 2350. Largo Eduardo Z. Faraco, Porto Alegre, RS, 90035-903, Brazil

## Abstract

**Introduction:**

Outcome after cardiac arrest is mostly determined by the degree of hypoxic brain damage. Patients recovering from cardiopulmonary resuscitation are at great risk of subsequent death or severe neurological damage, including persistent vegetative state. The early definition of prognosis for these patients has ethical and economic implications. The main purpose of this study was to investigate the prognostic value of serum neuron-specific enolase (NSE) in predicting outcomes in patients early after in-hospital cardiac arrest.

**Methods:**

Forty-five patients resuscitated from in-hospital cardiac arrest were prospectively studied from June 2003 to January 2005. Blood samples were collected, at any time between 12 and 36 hours after the arrest, for NSE measurement. Outcome was evaluated 6 months later with the Glasgow outcome scale (GOS). Patients were divided into two groups: group 1 (unfavorable outcome) included GOS 1 and 2 patients; group 2 (favorable outcome) included GOS 3, 4 and 5 patients. The Mann–Whitney *U *test, Student's *t *test and Fisher's exact test were used to compare the groups.

**Results:**

The Glasgow coma scale scores were 6.1 ± 3 in group 1 and 12.1 ± 3 in group 2 (means ± SD; *p *< 0.001). The mean time to NSE sampling was 20.2 ± 8.3 hours in group 1 and 28.4 ± 8.7 hours in group 2 (*p *= 0.013). Two patients were excluded from the analysis because of sample hemolysis. At 6 months, favorable outcome was observed in nine patients (19.6%). Thirty patients (69.8%) died and four (9.3%) remained in a persistent vegetative state. The 34 patients (81.4%) in group 1 had significantly higher NSE levels (median 44.24 ng/ml, range 8.1 to 370) than those in group 2 (25.26 ng/ml, range 9.28 to 55.41; *p *= 0.034).

**Conclusion:**

Early determination of serum NSE levels is a valuable ancillary method for assessing outcome after in-hospital cardiac arrest.

## Introduction

Since the introduction of closed-chest cardiac massage in 1960 [1] there have been several advances in cardiopulmonary resuscitation [2]. In spite of that, morbidity and mortality associated with cardiac arrest remain extremely high [3,4], with prognosis ranging from mild to moderate disability to persistent vegetative state. It is estimated that 80% of sudden death survivors remain in a coma for various lengths of time, and a full neurological recovery is still rare [5]. The possibility of irreversible anoxic brain damage must be taken into account soon after the arrest.

In this scenario, an accurate prognostic evaluation of cardiac arrest patients may have major ethical and economic consequences. Currently, prognosis is based on several clinical, neuroimaging and electrophysiological methods [6-9]. However, applying these methods is often difficult as a result of sedation and the hemodynamic instability commonly seen in critically ill patients. Biochemical markers, in contrast, are a low-cost alternative that may be more suitable for this purpose.

Neuron-specific enolase (NSE) is a known marker of ischemic brain damage and has already been evaluated in traumatic brain injury [10], stroke [11] and anoxic encephalopathy after cardiac arrest [12,13]. NSE, the neuronal form of the glycolytic enzyme enolase, is found almost exclusively in neurons and cells of neuroendocrine origin. It is a dimeric form compounded of two γ subunits that converts 2-phosphoglycerate into phosphoenolpyruvate, measurable in blood and cerebrospinal fluid [14].

As far as we know, there have been no studies focused on the prognostic value of NSE in patients surviving in-hospital cardiac arrest. The objective of this study was to prospectively evaluate the association of early NSE levels with patient outcome 6 months after in-hospital cardiac arrest, as measured by the Glasgow outcome scale (GOS) [15]. Our secondary goal was to establish a cutoff NSE level that could indicate unfavorable outcome (death or persistent vegetative state).

## Materials and methods

### Patients

We prospectively evaluated 45 patients who survived an in-hospital cardiac arrest in the period from June 2003 to January 2005 at the Hospital de Clínicas de Porto Alegre and the Complexo Hospitalar Santa Casa, two tertiary-care university hospitals in Porto Alegre, Brazil. We included patients who were successfully resuscitated after in-hospital cardiac arrest, as defined by the absence of palpable pulse and effective spontaneous ventilation with initial rhythm ventricular fibrillation, pulseless ventricular tachycardia, pulseless electrical activity and asystole, who survived for at least 12 hours after the event and for whom informed consent was obtained from the next of kin. The study was approved by the ethics committees of both hospitals. We excluded patients under the age of 16 years, those presenting drug intoxication, accidental or therapeutic hypothermia, those with neoplastic diseases known to increase NSE levels, stroke (ischemic and/or hemorrhagic) or traumatic brain injury in the previous 30 days, and patients subjected to extracorporeal circulation in the previous 30 days.

Patients were evaluated in terms of age, sex, duration of resuscitation efforts, Glasgow coma scale (GCS) score, pupillary reactivity to light, need of sedation, and time interval to blood sampling for NSE measurement. Resuscitation protocols followed American Heart Association guidelines [16]. Every resuscitated patient was admitted to an intensive care unit and the care provided followed the routine of the units, without interference from the investigators. Neurological examinations were performed together with blood sampling for NSE measurement between 12 and 36 hours after cardiac arrest. Attending physicians and the critical care team were unaware of the results of NSE measurements. None of the patients had a do-not-resuscitate order and there was no limitation of life support.

### Procedure

Blood samples were withdrawn by peripheral vein puncture and centrifuged for 10 minutes at 2,500 rotations per minute. Serum (1 ml) was frozen and stored at -86°C. Hemolyzed samples were considered lost. NSE measurements were performed with an electrochemiluminescence immunoassay (ECLIA), using a sandwich technique, in duplicate, with NSE kits (Roche, Mannheim, Germany) and the Elecsys 2010 analyzer (Roche Diagnostics, Mannheim, Germany). NSE measurements were also performed in seven control individuals.

The surviving patients were contacted by phone [17,18], 6 months after the date of the cardiac arrest, to evaluate neurological status measured by the GOS. The performance categories were defined as follows: GOS 1, death; GOS 2, persistent vegetative state; GOS 3, severe disability (unable to live independently, but capable of following commands); GOS 4, moderate disability (able to live independently, but unable to return to work); GOS 5, mild or no disability (able to return to work). For the purpose of this study, outcomes were separated into two groups: group 1 included patients who died or remained in a persistent vegetative state (GOS 1 and 2), and group 2 was formed by patients who recovered consciousness (GOS 3, 4 and 5). A patient was considered conscious if awake or capable of following simple commands at least once.

### Statistical analysis

Continuous data are presented as means and SD, and nonparametric data as medians and interquartile range. Student's *t *test and the Mann–Whitney *U *test were used to compare continuous data; Fisher's exact test was used to compare proportions. The discriminative power of NSE to predict an unfavorable outcome was determined by analysis of receiver-operating characteristics. The significance level was set at *p *< 0.05. Statistical analysis was performed with the Statistical Package for the Social Sciences (SPSS) version 12.0 (SPSS Inc., Chicago, IL, USA).

## Results

Of the 45 patients evaluated, two were excluded from the analysis because sample hemolysis prevented NSE measurement. Of the remaining 43 patients, 30 (69.8%) died (GOS 1) and four (9.3%) developed a persistent vegetative state (GOS 2). Thus, 34 patients were included in group 1. The outcome after 6 months was favorable (GOS 3, 4 and 5) in nine patients (20.9%), who were included in group 2. One of them survived with severe disability (GOS 3); eight survived with minimal disability (GOS 4 and 5).

The clinical characteristics of patients are shown in Table 1. The groups were similar in terms of age, sex, duration of resuscitation efforts, and need for sedation. The GCS score was significantly lower in group 1 than in group 2. All patients in group 2 presented pupillary reactivity to light, in contrast with 20 patients (59%) in group 1. This comparison was significantly different.

**Figure 1:**
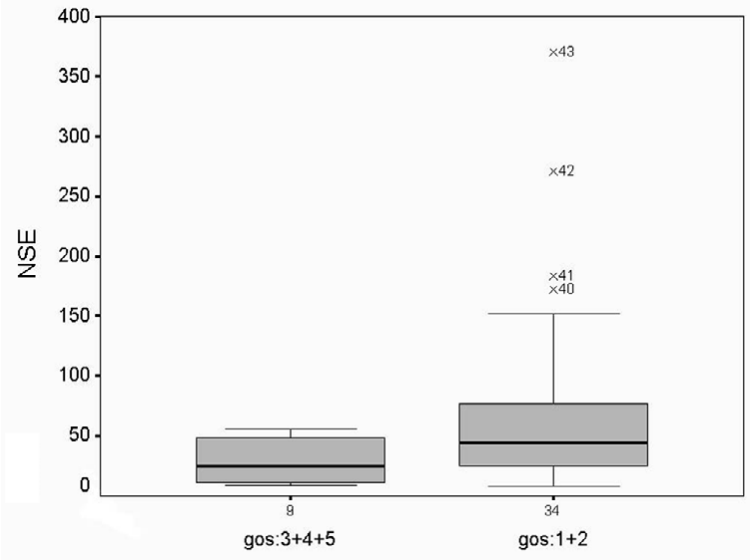
Neuron-specific enolase levels (ng/ml) after in-hospital cardiac arrest. Median, interquartile ranges and 5 to 95% centiles are shown. GOS, Glasgow outcome scale.

As shown in Figure 1, NSE levels measured between 12 and 36 hours were significantly higher in group 1 (median 44.24 ng/ml, range 8.1 to 370) than in group 2 (median 25.26 ng/ml, range 9.28 to 55.41; *p *= 0.034). NSE levels were significantly higher in group 2 patients (median 25.26 ng/ml, range 9.28 to 55.41) than in controls (median 9.34 ng/ml, range 8.39 to 10.53; *p *= 0.026).

**Figure 2:**
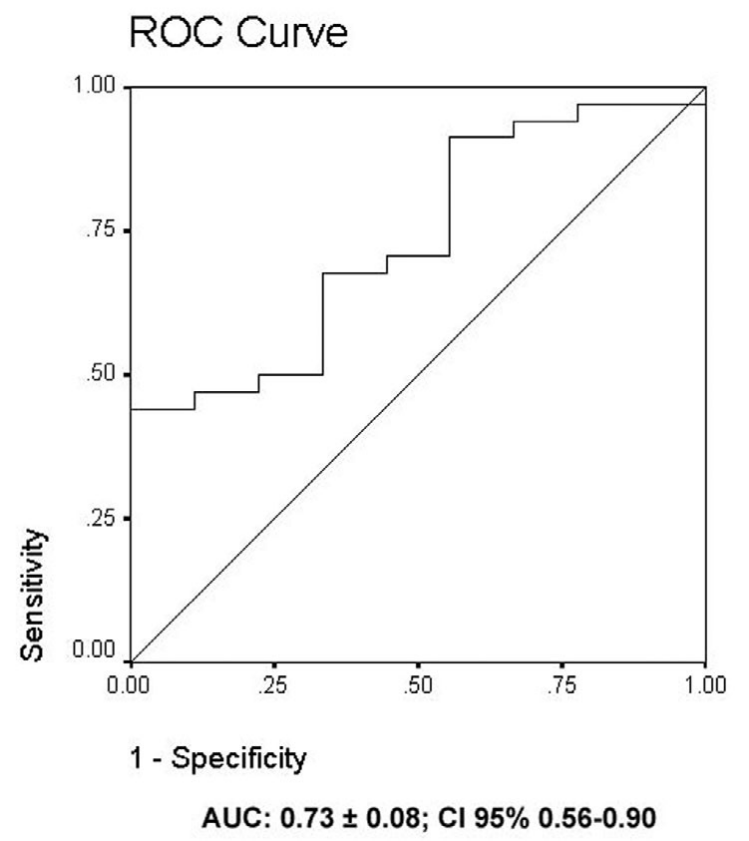
Receiver operating characteristics curve for neuron-specific enolase levels after in-hospital cardiac arrest. AUC, area under curve; CI, confidence interval.

The prognostic value of serum NSE in predicting unfavorable outcome was evaluated with a receiver operating characteristics curve. The area under the curve was 0.73 ± 0.08 (95% confidence interval (CI) 0.56 to 0.90; Figure 2). When a cutoff value of 60 ng/ml was established, a specificity of 100% (95% CI 66 to 100%) and a sensitivity of 35% (95% CI 19 to 53%) were obtained, with positive and negative predictive values of 100% (95% CI 73 to 100%) and 29% (95% CI 14 to 48%), respectively.

## Discussion

The most important finding of our study was the observation that increased NSE levels between 12 and 36 hours after in-hospital cardiac arrest are markers of ischemic brain damage and of unfavorable outcome. NSE levels measured early in the course of brain injury were significantly higher in patients with unfavorable outcomes (GOS 1 and 2) than in patients with favorable outcomes (GOS 3, 4 and 5) after 6 months.

Of the 43 patients analyzed after in-hospital cardiac arrest, 30 (69.8%) died and four (9.6%) remained in a persistent vegetative state. This mortality rate is in agreement with that described for other cohorts of in-hospital cardiac arrest. Peberdy and colleagues [19], for example, reported an 83% in-hospital mortality rate.

The GCS score was significantly lower in non-survivors and in patients who evolved to a persistent vegetative state than in those who survived after 6 months. Edgren and colleagues [20] have reported that absent motor response to pain and absent pupillary reactivity to light at 48 hours are good clinical parameters for the prediction of poor outcomes after global cerebral ischemia. The main limitation of performing a neurological examination in those patients is the need for sedation, which can grossly interfere with the evaluation.

It is known that NSE values are relatively low at the beginning of ischemic brain injury, with low predictive power in the first 6 hours. Böttiger and colleagues [21] were able to demonstrate prognostic usefulness only after 24 hours, and Rosén and colleagues [22] after 48 hours. In contrast, our study raised evidence that it is possible to establish prognosis at an earlier time. NSE measurements were made earlier in this study and samples were collected not at specific times but at any time between 12 and 36 hours. Although the absence of time course measurements could be a limitation, the fact that sampling does not need to be made at a defined time point greatly increases the clinical applicability of using NSE levels as a marker of prognosis after cardiac arrest, because this step can be included as part of the routine laboratory workup. In addition, our results show that we were able to maintain prognostic accuracy. As reported by Fogel and colleagues [23] and Schoerkhuber and colleagues [24], we observed significantly higher NSE levels in patients with poor outcome. Those authors, however, suggest that measurements be made after 72 hours, when NSE levels peak.

The difference in terms of time at NSE sampling between the groups, despite being a methodological limitation, is unlikely to have compromised the present results, because NSE has an ascending curve with peak values at about 72 to 96 hours [24,25]. Because sampling was performed earlier in group 1, we would probably have found an even greater difference between the two groups had the samples been collected at the same time.

To predict poor outcome in an individual patient, a highly specific marker is essential. The main reason for this is to avoid an unnecessarily pessimistic prognosis. For an NSE concentration of 60 ng/ml, a specificity of 100% and a sensitivity of 35% were obtained to indicate poor prognosis, with positive and negative predictive values of 100% and 29%, respectively. Twelve of the 43 patients studied had NSE levels above the cutoff point, and all of them died. Had NSE levels been used to make decisions about withholding or withdrawing critical care in these patients, there would have been a theoretical decrease of 63 days in the intensive care unit in this cohort. It should be noted that the proposed cutoff point was established retrospectively, and therefore requires further validation. Table 2 compares sensitivity and specificity and other relevant aspects in the present and previous studies [12,13,23-27].

Currently, the most accepted method for establishing prognosis in anoxic encephalopathy after cardiac arrest is the measurement of bilateral cortical response to somatosensory evoked potential (SSEP) [28], which is not widely available in our and other settings [23,25]. In contrast, determination of NSE levels can be done at low cost, is easily performed at the bedside and is not influenced by sedation, as occurs with neurological examination. In this study, 25% of the patients received sedatives. This makes the determination of NSE levels a very attractive ancillary prognostic method to be used after cardiopulmonary resuscitation. Zandbergen and colleagues [27] have recently shown that unfavorable outcome could be reliably predicted with both SSEP and NSE as early as 24 hours after a cardiac arrest in a cohort of 407 normothermic patients, most of whom were survivors of an out-of-hospital cardiac arrest. Using a predefined cutoff value of 33 ng/ml, NSE measurements were performed at least once in 231 patients and a 100% specificity was reached for unfavorable outcome, measured by the GOS a month after the event. Despite the fact that the results of SSEP and NSE overlapped only partly, those authors state that both tests were superior to all clinical tests.

Other biochemical markers have been studied to predict outcome after anoxic encephalopathy. S100 B is a protein originating in glial cells, in contrast with NSE, which is of neuronal origin. S100 B has been shown to be a good predictor of neurological recovery in patients surviving cardiac arrest [12,13,29], and it seems to have a good correlation with NSE in those patients [22]. High levels of creatinine kinase-BB isoenzyme in cerebrospinal fluid have also been associated with worse neurological outcome after ischemic brain damage [30].

Recently, therapeutic hypothermia has been shown to improve neurological outcomes in patients surviving cardiac arrest caused by ventricular fibrillation [31,32]. A recent study suggests that the use of therapeutic hypothermia reduces the prognostic value of NSE and S100 B to predict poor outcomes after cardiac arrest [29], which does not seem to happen with the use of evoked potentials [33].

The present results are not generalizable to a larger population of cardiac arrest cases, because we studied only in-hospital cardiac arrests. Nevertheless, these results are in agreement with, and complementary to, previous NSE studies with out-of-hospital cardiac arrest populations. A large prospective multicentric study to test a predefined cutoff value for NSE, using multiple samples and including patients treated with therapeutic hypothermia, surviving in-hospital and out-of-hospital arrests, should be performed before NSE measurements can be routinely used for decision-making about the maintenance of care in comatose patients after cardiac arrest.

## Conclusion

Our study demonstrates that NSE levels measured early in the course of ischemic cerebral injury are significantly higher in patients with unfavorable outcome than in patients with favorable outcome. Considering that prolonged cardiopulmonary resuscitation can produce irreversible anoxic brain damage, prognosis should be established as soon as possible. A multimodal approach combining several methods for prognostic evaluation, including neurological examination, electrophysiological studies and NSE measurements, should be used. We believe that this strategy may provide a more precise prognosis for these patients.

## Key messages

• Determination of serum neuron-specific enolase levels is a valuable ancillary method for assessing outcome after in-hospital cardiac arrest.

• Early serum neuron-specific enolase levels are higher in patients with unfavorable outcome 6 months after an in-hospital cardiac arrest.

## Abbreviations

CI = confidence interval; GCS = Glasgow coma scale; GOS = Glasgow outcome scale; NSE = neuron-specific enolase; SSEP = somatosensory evoked potential.

## Competing interests

The authors declare that they have no competing interests.

## Authors' contributions

THR conceived the project, participated in data collection, analysis and interpretation, and helped draft the manuscript. FN participated in data analysis. SRRV contributed to the study design and interpretation of data and revised the manuscript critically for important intellectual content. JSB provided intellectual input and contributed to study design and interpretation of results. RS performed measurements of serum NSE. All authors read and approved the final manuscript.

## Figures and Tables

**Table 1:** Baseline characteristics of 43 patients resuscitated from in-hospital cardiac arrest

Characteristic	Group 1 (GOS 1/2)	Group 2 (GOS 3–5)	*p*
*n*	34	9	-
Age, mean ± SD (years)	63.9 ± 14	64.7 ± 11	0.892
Male sex, *n *(percentage)	14 (41)	6 (66)	0.263
Initial rhythm			
VF/VT, *n *(percentage)	4 (12)	4 (44)	0.046
PEA/asystole, *n *(percentage)	30 (88)	5 (55)	
Low-flow time, median [range], (minutes)	11.5 [5–90]	13 [3–35]	0.952
GCS score, mean ± SD	6.1 ± 3	12.1 ± 3	< 0.001
Pupillary reaction to light, *n *(percentage)	20 (59)	9 (100)	0.020
Sedation use, *n *(percentage)	10 (29)	1 (11)	0.407
Δ*t *from NSE sampling, mean ± SD (hours)	20.2 ± 8.3	28.4 ± 8.7	0.013

GOS, Glasgow outcome scale; VF, ventricular fibrillation; VT, ventricular tachycardia; PEA, pulseless electrical activity; GCS, Glasgow Coma Scale; Δ*t*, time elapsed from cardiopulmonary resuscitation until blood sampling for NSE measurement; NSE, neuron-specific enolase.

**Table 2:** Studies of serum neuron-specific enolase to predict unfavorable outcome after cardiac arrest

Reference	In-hospital CPR	NSE sampling time (hours)	Favorable outcome (*n*)	Unfavorable outcome (*n*)	Cut-off value (ng/ml)	Sensitivity (percentage)	Specificity (percentage)
[26]	No	24	45	20	>17	40	98
[23]	Not specified	24	27	35	>20	51	89
[12]	No	72	18	25	>33	65	100
[24]	No	72	28	24	>16.4	70	100
[25]	Yes/No^a^	48	34	76	>25	59	100
[13]	Yes/No^b^	72	28	69	>65	50	96
[27]	Yes/No^c^	24	51^d^	356^d^	>33	44	100
This study	Yes	12–36	34	9	>60	35	100

CPR = cardiopulmonary resuscitation; NSE = neuron-specific enolase. ^a^77% out-of-hospital arrests; ^b^56% were out-of-hospital arrests; ^c^85% were out-of-hospital arrests; ^d^NSE levels were determined in 231 of 407 patients.
